# Poly[*trans*-diaqua­bis[μ-3-(3-pyrid­yl)propionato-κ^2^ *N*,*O*]cadmium(II)]

**DOI:** 10.1107/S1600536810005222

**Published:** 2010-02-13

**Authors:** Young Ok Jang, Soon W. Lee

**Affiliations:** a Department of Chemistry (BK21), Sungkyunkwan University, Natural Science Campus, Suwon 440-746, Republic of Korea

## Abstract

The title compound [Cd(*L*)_2_(H_2_O)_2_]_*n*_ (*L* = 3-pyridine­propionic acid, C_8_H_8_NO_2_), is a two-dimensional coordination polymer in which the Cd^II^ ion lies on an inversion center and is coordinated in a slightly distorted octa­hedral environment. The aqua H atoms are involved in inter­molecular O–H⋯O hydrogen bonds, which extend the two-dimensional structure to a three-dimensional architecture. The Cd⋯Cd separation within a layer is 9.0031 (1) Å.

## Related literature

For the isostructural zinc analog, see: Wang *et al.* (2006 ▶) and for the cobalt and nickel analogs, see: Martin *et al.* (2007 ▶). For background information on coordination polymers, see: Batten *et al.* (2009 ▶); Lu (2003 ▶); Perry *et al.* (2009 ▶); Robin & Fromm (2006 ▶). For coordination polymers based on pyridine carboxyl­ates, see: Huh & Lee (2006 ▶, 2007 ▶, 2008 ▶); Kim *et al.* (2007 ▶); Min *et al.* (2001 ▶, 2002 ▶); Min & Lee (2002 ▶). 
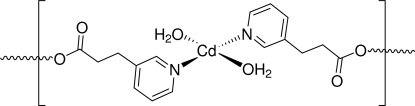


## Experimental

### 

#### Crystal data

[Cd(C_8_H_8_NO_2_)_2_(H_2_O)_2_]
*M* *_r_* = 448.74
Monoclinic, 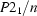

*a* = 9.6934 (4) Å
*b* = 8.9082 (4) Å
*c* = 10.1199 (5) Å
β = 104.309 (2)°
*V* = 846.75 (7) Å^3^
*Z* = 2
Mo *K*α radiation
μ = 1.33 mm^−1^
*T* = 296 K
0.42 × 0.38 × 0.28 mm

#### Data collection

Bruker SMART CCD diffractometer
Absorption correction: multi-scan (*SADABS*; Sheldrick, 1996 ▶) *T*_min_ = 0.606, *T*_max_ = 0.708
12941 measured reflections
2113 independent reflections
1911 reflections with *I* > 2σ(*I*)
*R*_int_ = 0.021

#### Refinement

*R*[*F* ^2^ > 2σ(*F* ^2^)] = 0.016
*wR*(*F* ^2^) = 0.042
*S* = 1.05
2113 reflections
155 parameters
All H-atom parameters refined
Δρ_max_ = 0.30 e Å^−3^
Δρ_min_ = −0.22 e Å^−3^

### 

Data collection: *SMART* (Bruker, 1997 ▶); cell refinement: *SAINT* (Bruker, 1997 ▶); data reduction: *SAINT*; program(s) used to solve structure: *SHELXTL* (Sheldrick, 2008 ▶); program(s) used to refine structure: *SHELXTL*; molecular graphics: *SHELXTL* software used to prepare material for publication: *SHELXTL*.

## Supplementary Material

Crystal structure: contains datablocks global, I. DOI: 10.1107/S1600536810005222/lh2993sup1.cif

Structure factors: contains datablocks I. DOI: 10.1107/S1600536810005222/lh2993Isup2.hkl

Additional supplementary materials:  crystallographic information; 3D view; checkCIF report

## Figures and Tables

**Table:** 

Cd1—O3^i^	2.2704 (9)
Cd1—O1	2.3306 (11)
Cd1—N1	2.3374 (10)

**Table:** 

O3^i^—Cd1—O1	86.35 (4)
O3^i^—Cd1—N1^ii^	91.31 (4)
O1—Cd1—N1	89.58 (4)

Symmetry codes: (i) 
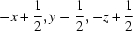
; (ii) 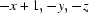.

**Table 2:** Hydrogen-bond geometry (Å, °)

*D*—H⋯*A*	*D*—H	H⋯*A*	*D*⋯*A*	*D*—H⋯*A*
O1—H*O*1*B*⋯O2^iii^	0.83 (3)	2.01 (3)	2.8361 (16)	174 (2)
O1—H*O*1*A*⋯O2^iv^	0.88 (2)	1.94 (2)	2.7546 (17)	155 (2)

Symmetry codes: (iii) 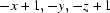; (iv) 
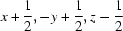
.

**Fig. 1.**
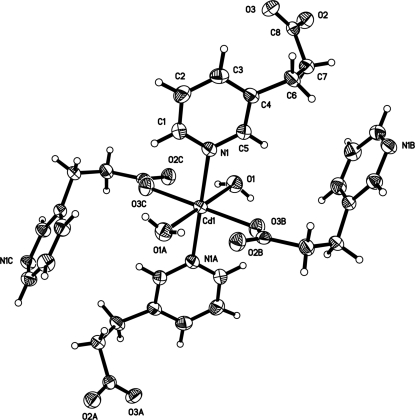
Local coordination environment around the Cd metal in the title compound showing 50% probability displacement ellipsoids (symmetry codes: (A) -x + 1, -y, -z, (B) -x + 1/2, y - 1/2, -z + 1/2, (C) x + 1/2, -y + 1/2, z - 1/2.

**Fig. 2.**
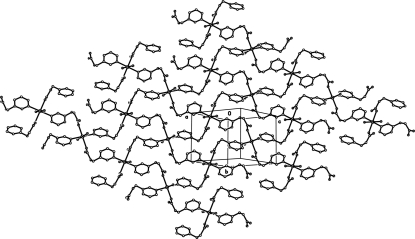
Packing diagram of the title compound, showing a two-dimensional network.

## supplementary crystallographic information

### Comment

Coordination polymers have gained attention due to their desirable properties applicable to size-selective adsorption, gas storage, host–guest recognition, catalysis, and photoluminescence (Batten *et al.*, 2009; Perry IV *et al.*, 2009; Robin & Fromm, 2006). We have been continually interested in the preparation, structures, and properties of coordination polymers based on the linking ligands of pyridine carboxylate derivatives, in which the carboxylate groups are directly attached to the pyridine ring (Huh & Lee, 2006; Huh & Lee, 2007; Huh & Lee, 2008; Kim *et al.*, 2007; Min *et al.*, 2001; Min *et al.*, 2002; Min & Lee, 2002).

Linking ligands containing both N-donors and O-donors are frequently used for the construction of coordination polymers (Lu, 2003). In particular, silver, copper, zinc, cobalt, and nickel coordination polymers containing 3-pyridinepropionic acid as a linking ligand have been reported (Wang *et al.*, 2006; Martin *et al.*, 2007). This ligand has an enthylene (–CH_2_–CH_2_–) spacer between the pyridyl and carboxylate groups and therefore is flexible. As an extension of our study, we investigated the preparation of cadmium coordination polymers by employing this ligand.

The title compound is isostructural with the zinc (Wang *et al.*, 2006), cobalt, and nickel (Martin *et al.*, 2007) analogs with the empirical formula [Cd(L)_2_(H_2_O)_2_] (L = 3-pyridinepropionic acid). The local coordination environment around the Cd atom and the atom-numbering scheme is shown in Fig. 1. The asymmetric unit consists of one half Cd^II^ ion, one L ligand, and one aqua ligand. The Cd^II^ ion lies on a crystallographic inversion center, and the remaining atoms occupy general positions. The coordination environment of the Cd^II^ ion is slightly-distorted octahedral. The monomer units [Cd(L)_2_(H_2_O)_2_] are linked by covalent bonds (Cd–N and Cd–O) to form a 2-D layer approximately in the (101) plane (Fig. 2) and then extended into a 3-D architecture by hydrogen bonding. Two carboxylate O atoms (O2 and O3) act differently. Whereas O2 acts as a H-bond acceptor to the aqua ligands in the neighboring units, O3 is coordinated to the Cd metal to contribute to the formation of the 2-D layer, in which the Cd···Cd separation is 9.0031 (1) Å.

### Experimental

A mixture of 3-pyridinepropionic acid (0.98 g, 6.5 mmol), [Cd(NO_3_)_2_]6(H_2_O) (1 g, 3.2 mmol), NaOH (6.5 mmol), and H_2_O (6 ml) was heated at 453 K for 2 days in a 23 ml Teflon-lined stainless-steel autoclave and then cooled slowly to room temperature to obtain pale yellow crystals. The product was collected by filtration, washed with H_2_O (3 × 10 ml) and ethanol (5 × 10 ml), and then air-dried. (1.18 g, 2.6 mmol, 82%). mp: 538–540 K. IR (KBr, cm^-1^): 3199 (*s*), 2173 (w), 1606 (*s*), 1302 (w), 1247 (w), 1201 (w), 1117 (*m*), 1047 (*m*), 961 (*s*), 607 (*s*)

### Refinement

All H atoms were located and refined isotropically.

### Crystal data

**Table:**

[Cd(C_8_H_8_NO_2_)_2_(H_2_O)_2_]	*F*(000) = 452
*M**_r_* = 448.74	*D*_x_ = 1.760 Mg m^−^^3^
Monoclinic, *P*2_1_/*n*	Mo *K*α radiation, λ = 0.71073 Å
Hall symbol: -P 2yn	Cell parameters from 8598 reflections
*a* = 9.6934 (4) Å	θ = 2.6–28.4°
*b* = 8.9082 (4) Å	µ = 1.33 mm^−^^1^
*c* = 10.1199 (5) Å	*T* = 296 K
β = 104.309 (2)°	Block, colourless
*V* = 846.75 (7) Å^3^	0.42 × 0.38 × 0.28 mm
*Z* = 2	

### Data collection

**Table:**

Bruker SMART CCD diffractometer	2113 independent reflections
Radiation source: sealed tube	1911 reflections with *I* > 2σ(*I*)
graphite	*R*_int_ = 0.021
φ and ω scans	θ_max_ = 28.4°, θ_min_ = 3.1°
Absorption correction: multi-scan (*SADABS*; Sheldrick, 1996)	*h* = −12→12
*T*_min_ = 0.606, *T*_max_ = 0.708	*k* = −11→11
12941 measured reflections	*l* = −13→13

### Refinement

**Table:**

Refinement on *F*^2^	Primary atom site location: structure-invariant direct methods
Least-squares matrix: full	Secondary atom site location: difference Fourier map
*R*[*F*^2^ > 2σ(*F*^2^)] = 0.016	Hydrogen site location: inferred from neighbouring sites
*wR*(*F*^2^) = 0.042	All H-atom parameters refined
*S* = 1.05	*w* = 1/[σ^2^(*F*_o_^2^) + (0.0233*P*)^2^ + 0.1949*P*] where *P* = (*F*_o_^2^ + 2*F*_c_^2^)/3
2113 reflections	(Δ/σ)_max_ = 0.001
155 parameters	Δρ_max_ = 0.30 e Å^−^^3^
0 restraints	Δρ_min_ = −0.22 e Å^−^^3^

### Special details

**Table:**

Geometry. All esds (except the esd in the dihedral angle between two l.s. planes) are estimated using the full covariance matrix. The cell esds are taken into account individually in the estimation of esds in distances, angles and torsion angles; correlations between esds in cell parameters are only used when they are defined by crystal symmetry. An approximate (isotropic) treatment of cell esds is used for estimating esds involving l.s. planes.
Refinement. Refinement of *F*^2^ against ALL reflections. The weighted *R*-factor wR and goodness of fit *S* are based on *F*^2^, conventional *R*-factors *R* are based on *F*, with *F* set to zero for negative *F*^2^. The threshold expression of *F*^2^ > σ(*F*^2^) is used only for calculating *R*-factors(gt) etc. and is not relevant to the choice of reflections for refinement. *R*-factors based on *F*^2^ are statistically about twice as large as those based on *F*, and *R*- factors based on ALL data will be even larger.

### Fractional atomic coordinates and isotropic or equivalent isotropic displacement parameters (Å^2^)

**Table:**

	*x*	*y*	*z*	*U*_iso_*/*U*_eq_	
Cd1	0.5000	0.0000	0.0000	0.02437 (5)	
N1	0.28876 (11)	0.07564 (12)	0.05233 (11)	0.0301 (2)	
O1	0.61972 (13)	0.02896 (15)	0.22844 (12)	0.0382 (2)	
O2	0.21558 (10)	0.21211 (11)	0.63186 (10)	0.0359 (2)	
O3	0.04577 (10)	0.26152 (10)	0.44405 (10)	0.0352 (2)	
C1	0.20346 (15)	0.17483 (15)	−0.02640 (14)	0.0329 (3)	
C2	0.07951 (16)	0.22538 (17)	0.00161 (16)	0.0379 (3)	
C3	0.04061 (15)	0.16952 (16)	0.11451 (15)	0.0357 (3)	
C4	0.12544 (13)	0.06336 (15)	0.19621 (13)	0.0284 (3)	
C5	0.24972 (16)	0.02120 (15)	0.16092 (16)	0.0303 (3)	
C6	0.08326 (19)	−0.01072 (15)	0.31409 (17)	0.0345 (3)	
C7	0.17242 (17)	0.03087 (17)	0.45527 (15)	0.0325 (3)	
C8	0.14245 (13)	0.18100 (13)	0.51422 (13)	0.0260 (2)	
H1	0.2335 (18)	0.208 (2)	−0.1008 (18)	0.042 (4)*	
H2	0.0224 (18)	0.296 (2)	−0.0590 (18)	0.043 (4)*	
H3	−0.0467 (19)	0.199 (2)	0.1376 (18)	0.050 (5)*	
H5	0.314 (2)	−0.048 (2)	0.2165 (19)	0.044 (5)*	
H6A	−0.022 (2)	0.0146 (17)	0.302 (2)	0.045 (6)*	
H6B	0.0908 (17)	−0.1217 (19)	0.3076 (17)	0.039 (4)*	
H7A	0.274 (3)	0.037 (3)	0.460 (2)	0.059 (6)*	
H7B	0.158 (2)	−0.041 (2)	0.525 (2)	0.045 (5)*	
HO1A	0.673 (2)	0.105 (3)	0.217 (2)	0.066 (7)*	
HO1B	0.672 (3)	−0.041 (3)	0.266 (3)	0.065 (7)*	

### Atomic displacement parameters (Å^2^)

**Table:**

	*U*^11^	*U*^22^	*U*^33^	*U*^12^	*U*^13^	*U*^23^
Cd1	0.02423 (8)	0.02733 (8)	0.02284 (8)	−0.00074 (4)	0.00826 (5)	−0.00185 (4)
N1	0.0303 (5)	0.0323 (6)	0.0310 (6)	0.0030 (4)	0.0137 (4)	0.0022 (4)
O1	0.0427 (6)	0.0401 (6)	0.0278 (5)	−0.0014 (5)	0.0011 (5)	−0.0019 (4)
O2	0.0375 (5)	0.0343 (5)	0.0338 (5)	0.0007 (4)	0.0047 (4)	−0.0022 (4)
O3	0.0369 (5)	0.0296 (5)	0.0372 (5)	0.0071 (4)	0.0055 (4)	−0.0010 (4)
C1	0.0388 (7)	0.0314 (6)	0.0303 (7)	0.0007 (5)	0.0120 (6)	0.0035 (5)
C2	0.0378 (7)	0.0357 (7)	0.0387 (8)	0.0090 (6)	0.0064 (6)	0.0053 (6)
C3	0.0293 (6)	0.0380 (7)	0.0413 (8)	0.0047 (5)	0.0119 (6)	−0.0040 (6)
C4	0.0309 (6)	0.0288 (6)	0.0281 (6)	−0.0039 (5)	0.0120 (5)	−0.0049 (5)
C5	0.0317 (7)	0.0316 (7)	0.0295 (7)	0.0043 (5)	0.0114 (6)	0.0036 (5)
C6	0.0407 (8)	0.0357 (8)	0.0318 (8)	−0.0082 (5)	0.0178 (6)	−0.0043 (5)
C7	0.0385 (8)	0.0323 (6)	0.0286 (7)	0.0063 (6)	0.0120 (6)	0.0010 (5)
C8	0.0265 (6)	0.0252 (6)	0.0294 (6)	−0.0013 (4)	0.0126 (5)	0.0024 (5)

### Geometric parameters (Å, °)

**Table:**

Cd1—O3^i^	2.2704 (9)	C1—H1	0.919 (18)
Cd1—O3^ii^	2.2705 (9)	C2—C3	1.381 (2)
Cd1—O1	2.3306 (11)	C2—H2	0.954 (17)
Cd1—O1^iii^	2.3306 (11)	C3—C4	1.385 (2)
Cd1—N1^iii^	2.3374 (10)	C3—H3	0.969 (18)
Cd1—N1	2.3374 (10)	C4—C5	1.3902 (18)
N1—C1	1.3318 (17)	C4—C6	1.5055 (19)
N1—C5	1.3385 (18)	C5—H5	0.96 (2)
O1—HO1A	0.88 (2)	C6—C7	1.522 (2)
O1—HO1B	0.83 (3)	C6—H6A	1.03 (2)
O2—C8	1.2568 (15)	C6—H6B	0.995 (17)
O3—C8	1.2522 (15)	C7—C8	1.5213 (19)
O3—Cd1^iv^	2.2705 (9)	C7—H7A	0.98 (2)
C1—C2	1.3766 (19)	C7—H7B	0.99 (2)
			
O3^i^—Cd1—O3^ii^	180.0	C1—C2—H2	118.7 (11)
O3^i^—Cd1—O1	86.35 (4)	C3—C2—H2	122.4 (11)
O3^ii^—Cd1—O1	93.65 (4)	C2—C3—C4	119.78 (12)
O3^i^—Cd1—O1^iii^	93.65 (4)	C2—C3—H3	122.3 (11)
O3^ii^—Cd1—O1^iii^	86.35 (4)	C4—C3—H3	117.9 (11)
O1—Cd1—O1^iii^	180.00 (3)	C3—C4—C5	117.09 (12)
O3^i^—Cd1—N1^iii^	91.31 (4)	C3—C4—C6	122.34 (12)
O3^ii^—Cd1—N1^iii^	88.69 (4)	C5—C4—C6	120.49 (13)
O1—Cd1—N1^iii^	90.42 (4)	N1—C5—C4	123.49 (13)
O1^iii^—Cd1—N1^iii^	89.58 (4)	N1—C5—H5	116.2 (11)
O3^i^—Cd1—N1	88.69 (4)	C4—C5—H5	120.3 (11)
O3^ii^—Cd1—N1	91.31 (4)	C4—C6—C7	115.77 (12)
O1—Cd1—N1	89.58 (4)	C4—C6—H6A	105.5 (12)
O1^iii^—Cd1—N1	90.42 (4)	C7—C6—H6A	112.2 (13)
N1^iii^—Cd1—N1	180.00 (5)	C4—C6—H6B	110.2 (10)
C1—N1—C5	118.16 (11)	C7—C6—H6B	105.7 (9)
C1—N1—Cd1	120.32 (9)	H6A—C6—H6B	107.3 (13)
C5—N1—Cd1	121.52 (9)	C8—C7—C6	117.58 (12)
Cd1—O1—HO1A	97.1 (14)	C8—C7—H7A	102.6 (13)
Cd1—O1—HO1B	118.0 (18)	C6—C7—H7A	113.1 (14)
HO1A—O1—HO1B	109 (2)	C8—C7—H7B	102.8 (12)
C8—O3—Cd1^iv^	124.02 (8)	C6—C7—H7B	111.3 (12)
N1—C1—C2	122.56 (13)	H7A—C7—H7B	108.6 (17)
N1—C1—H1	115.2 (11)	O3—C8—O2	125.36 (12)
C2—C1—H1	122.3 (11)	O3—C8—C7	118.03 (12)
C1—C2—C3	118.88 (13)	O2—C8—C7	116.59 (11)

Symmetry codes: (i) −*x*+1/2, *y*−1/2, −*z*+1/2; (ii) *x*+1/2, −*y*+1/2, *z*−1/2; (iii) −*x*+1, −*y*, −*z*; (iv) −*x*+1/2, *y*+1/2, −*z*+1/2.

### Hydrogen-bond geometry (Å, °)

**Table:**

*D*—H···*A*	*D*—H	H···*A*	*D*···*A*	*D*—H···*A*
O1—HO1B···O2^v^	0.83 (3)	2.01 (3)	2.8361 (16)	174 (2)
O1—HO1A···O2^ii^	0.88 (2)	1.94 (2)	2.7546 (17)	155 (2)

Symmetry codes: (v) −*x*+1, −*y*, −*z*+1; (ii) *x*+1/2, −*y*+1/2, *z*−1/2.
